# Testing Water for Organic Impurities

**Published:** 1869-01

**Authors:** 


Testing Water for Organic Impurities.—Half fill a common water bottle, cover its mouth with the hand, violently shake for a minute, and quickly apply to the nose. If nothing unpleasant is detected, tightly cork the bottle, set it in a warm place about the temperature of one's body for a couple or three days, and repeat the shaking. Water of very bad quality may thus be recognized without the trouble and expense of analysis.

